# Entrepreneurship in climate governance at the local and regional levels: concepts, methods, patterns, and effects

**DOI:** 10.1007/s10113-018-1351-5

**Published:** 2018-05-09

**Authors:** Dave Huitema, Elin Lerum Boasson, Raoul Beunen

**Affiliations:** 1 Institute for Environmental Studies (IVM), Vrije Universiteit Amsterdam, De Boelelaan 1087, 1081 HV Amsterdam, The Netherlands; 2 Department of Natural Sciences, Faculty of Management, Science and Technology, Open University of the Netherlands, Valkenburgerweg 177, 6419 AT Heerlen, the Netherlands; 3 Department of Political Science, University of Oslo, Moltke Moesvei 31, 0851 Oslo, Norway; 4 Centre for International Climate and Environmental Research (CICERO), Gaustadalléen 21, 0349 Oslo, Norway

**Keywords:** Climate governance, Entrepreneurship, Climate policy, Entrepreneurial strategies

## Abstract

This editorial sets the scene for a special issue on climate governance entrepreneurship at the local and regional levels. To make climate governance work, much policy activity is needed at the local and regional levels. Entrepreneurs are actors who aim to affect change by using their agency. They target policy decisions at the local and regional levels, which might subsequently turn to other governance levels to expand their influence. The scientific discussion about governance entrepreneurs is characterized by a lack of conceptual clarity, by methodological challenges, and by several research gaps. Regarding the latter, at present, it is especially unclear when and why entrepreneurs become active, which factors they take into account when they select their strategies, and what explains the effects of entrepreneurial activity on the emergence of innovations in climate governance. All contributions to this special issue engage with one or several of these conceptual, methodological, and empirical challenges, thus advancing the state of art in the field. Highlights from the special issue include the development of a simple conceptual frame that connects actors, contexts, strategies, and outcomes in a systematic way. Some promising methodological avenues are described, since the special issue contains not only some qualitative case studies but also some studies that take a long-term perspective by following policy development for decades, and a study that proposes a census approach. Empirically, the contributions in this special issue shed light on a range of factors explaining levels of entrepreneurial activity, and they carefully trace impacts over time. We conclude by sketching an agenda for further work in this realm.

## Paris, polycentric governance, and agency

The Paris agreement on climate change of 2015 possibly presents a watershed moment in the short and turbulent history of climate change governance. To many, the agreement seems like a great step forward—we now have almost universal global agreement on climate change targets (including the 1.5 degrees goal), after years of stalemate and disagreement. Quite a few observers have noted that the Paris agreement contains another big change: no longer will attempts be made—at the UN level that is—to agree on binding targets for individual countries. Instead, countries can now come forward—at their own initiative—with pledges on how much they will contribute to reaching the global goal, and these pledges will be submitted to a continuously revolving review cycle (“pledge and review”). And finally, in a related attempt to share responsibility and stimulate bottom-up initiative, local and regional governments are now explicitly recognized as key partner in addressing climate change, together with business and civil society.

In this new polycentric reality of climate governance, there are bound to be more opportunities for distributed local and regional actions (see also Green 2013, Widerberg and Pattberg 2014, and Bulkeley et al. 2014). The cumulative impact of these actions depends on various factors (see Jordan et al. 2018), including the degree to which climate governance initiatives will take off and prosper at a local level, mutual adjustment between initiatives, willingness to experiment and hence test ideas and evaluate their results, trust-building and overarching rules, such as goals to be achieved, and arrangements for conflict resolution (Ostrom 2010; Jordan et al. 2018).

Regardless of the expectations one has of these various developments, it is hard to contest that a *high level of initiative from the local and regional levels* is needed to make such a system function and so questions related to *agency* abound (Boasson 2018). It is useful to zoom in in governance entrepreneurship when we aim to enhance our understanding of questions such as the following: How do communities get going? Where do communities get their ideas from? How do they build the required networks and coalitions to break down established routines and cut to size incumbent interests? How do they evaluate their initiatives? And how do they connect to other communities and governance levels, compare notes, and exchange lessons with other communities? Who will take the initiative to develop overarching norms and standards, to create mechanisms to resolve conflicts, and bring such conflicts to the attention of for instance courts?

Local climate action will certainly require a lot of effort, time, and resources. One obvious question to ask here is whether there will be support from national governments. After all, some governments aim to obstruct climate action. But even if governments support local initiatives and subsequently are willing to scale up best practices, how would such a process be properly aligned with the fact that different local communities might have completely different preferences in terms of climate governance? Another challenge is that some groups and communities might have the ability to initiate climate governance initiatives, while others do not.

The focus of this special issue is on a special kind of actors in these processes, namely *governance entrepreneurs*, and we specifically focus on those operating at the local and regional levels. In the next section, we will elaborate a bit more on this concept, before we start outlining the research agenda for this special issue.

## Governance entrepreneurs at the local and regional levels

The term entrepreneur refers to a person taking action or to take the initiative (“entre prendere” means to undertake), and in common language, it is usually associated with business and the private sector (business entrepreneurs). If we follow Schumpeter’s famous words on the functioning of the market place, entrepreneurship is all about “creative destruction,” with new entrepreneurs constantly challenging the established ways of doing, and replacing them with new approaches that are more profitable. The notion that entrepreneurs also exist and partake in public matters (governing, or governance) is relatively new and is often attributed to John Kingdon (1984), who studied decision-making in Washington, DC, and found what he called *policy entrepreneurs* paving the way for changes in American federal policy. In Kingdon’s (1984) conceptualization, policy entrepreneurs are intrinsically motivated and persistent individuals who spend an extensive amount of resources in gathering support for their plans.

The thinking on policy entrepreneurs as developed by Kingdon (1984) was picked up in multiple places and has also spurred a small but fertile stream of analyses in the field of environmental governance. We mention a few relevant examples in the context of environmental issues, in order to demonstrate which kind of analyses can be made, and to show that this type of analysis can be applied at multiple jurisdictional levels. Young (1991), in seeking to understand the formation of global environmental regimes, regards entrepreneurial activity as a specific form of leadership. He suggested that the main role of entrepreneurial leaders is to draw attention to the character and importance of the issues at stake, to propose innovative policy solutions, and to broker compromises. Entrepreneurship, according to him, involves effectively setting or at least shaping the (climate policy) agenda and facilitating the adoption of particular solutions in specific interactions is a matter for entrepreneurial leadership, and thus, the entrepreneurial form of leadership is about diplomatic, negotiating, and bargaining skills. Entrepreneurial capacities are available to state actors and to non-state actors working in the climate regime, since both types of actors can engage in networking, alliance building, and compromise formation—although resources will vary. Boasson and Wettestad (2014) analyzed the insertion of carbon capture and storage (CCS) in climate policies of the European Union. They suggested that a coalition of actors with a long-term record of working on this approach with actors who had a key economic interest (certain oil companies), and politicians who were anxious to score political points in the short term, ensured that this approach was included and that funding became available for research and experiments with the approach. Their analysis shows that not all governance entrepreneurs need to have a long-term commitment to an issue or solution. Huitema and Meijerink (2009) finally analyzed 16 instances of major water policy change around the globe (the analysis included a combination of regional and national cases in China, the USA, India, South Africa, etc.). They concluded that the people they called policy entrepreneurs were consistently involved in the introduction of new policies, and that their strategies revolved around idea development (e.g., by experimenting and piloting), coalition building (entrepreneurship as a group process), venue shopping and creation (moving discussions to other or new jurisdictions), networking (making sure how other actors think), and framing (making sure problems get interpreted in a certain way).

In this special issue, we refer to *governance entrepreneurs* rather than policy entrepreneurs. What is *entrepreneurial* about governance entrepreneurs and why do we use the term governance? To answer the first question, it is clear that governance entrepreneurs are active actors, who meddle in the business of climate governance. Climate governance entrepreneurs are specifically interested in changing governance arrangements, for instance by creating new distributions of authority and information; by spreading new norms and cognitive frameworks, worldviews, and institutional logics; or by influencing (and sometimes blocking) the proposals that are made to that effect (Boasson and Huitema 2017). To answer the second question, our use of the term *governance* reflects the situation that academic and societal thinking on the role of government (which makes public policy—the type of policy studied by Kingdon) has evolved. In short, the provision of public and quasi-public goods and services is increasingly placed in the hands of the private sector and civil society. On top of that, many national governments are deeply entrenched in processes of decentralization, judicialization, Europeanization, or globalization and so the task of collective problem solving is increasingly shared between multiple types of actors, at multiple levels.

That we speak of governance does not mean that we regard the narrower public policy term irrelevant. In fact, national governments in both developed and developing countries have adopted an increasing number of climate measures and regulations (Townshend et al. 2013). This means that even if some climate measures are provided by private parties, they must ensure delivery does meet certain public standards. Another reason why the term policy still holds currency is that private parties also make them. Green (2013) for instance points out how large corporations such as Walmart can initiate significant corporate climate policy. She also suggests that public and private policies may go hand in hand and reinforce each other (private regulations pave the way and create certification standards for instance that can then be formally required by states; the contemplation of new public policies can motivate private parties to pick up issues themselves, etc.).

In an empirical sense, in this special issue, we are specifically interested in climate governance at the local and regional levels, pertaining to both the issues of mitigation and adaptation. Indeed, it has been argued, especially in the literature on the role of local and regional governments in climate governance (Bulkeley and Castán Broto, 2014; Hoffmann, 2011), that policy entrepreneurs can entice local governments into climate sensitive positions and can even turn them into proponents of climate measures globally. Local and regional entrepreneurs have aimed to change policy at the local and regional levels, or—after affecting local and regional governance—in turn they have sought to expand their influence to the national, or even global, levels. In this special issue, we will see that local and regional actors have developed novel approaches to climate change (e.g., setting new goals, inventing new means), and how they have tried to diffuse them more widely (by getting others to adopt them), and how they have tried to ensure that these approaches have an impact (Jordan and Huitema, 2014a, b).

Not all individuals, companies, localities, or regions will be motivated to engage with climate change and innovations in climate change governance. Whether or not they will do so,will depend—in part—on political preferences, on capabilities for self-organization, on knowledge and expertise, on the existence of networks with connection to the wider climate governance community, etc. But even between those actors who do feel compelled to seek change, there are indications that a qualitative difference exists between actors. Some will initiate climate action only if this is what they are required to by formal rules or feel socially obliged to, while others may aim to punch about their weight in their efforts to shape climate governance (Green 2017, Boasson and Huitema 2017).

One of the most interesting questions in entrepreneurship studies relates to why some actors perform entrepreneurship and others not, and why some entrepreneurs are more successful than others. Although we should not forget that sometimes entrepreneurs must also work *against* certain policy changes (for instance, initiatives that seek to dismantle climate governance arrangements), and we must keep in mind that even successful entrepreneurs will not always succeed, for instance when the odds are simply stacked against them.

## Guiding questions

So far, the literature on entrepreneurship in environmental governance has focused quite heavily on *successful entrepreneurs* (see Huitema and Meijerink 2009; Boasson and Wettestad 2014). Typically, this means tracing back the roots of visible policy changes and sorting out who was behind the change. Most headway has been made around the issue of the *strategies* that entrepreneurs use to advance their goals. It has been suggested that the strategies include the development of new ideas which can serve as an alternative to the current ways of addressing climate change, the experimentation and piloting with such novel approaches to (compare McFadgen and Huitema 2017), the deliberate framing of events, continuous networking to remain aware of the thinking of other actors, coalition building, and moving discussions to the most receptive venue (see Huitema and Meijerink 2009). Some authors have argued that entrepreneurs applying these strategies are capable of creating links between different levels and spheres of climate governance, ensuring that climate action in one domain influences governance initiatives elsewhere (see Boasson 2015). But despite the headway made, quite a few challenges remain and we hope this special issue is a contribution in addressing some of those. We see challenges at the c*onceptual*, at the *methodological*, and at the *empirical* levels.

The first set of challenges is at the *conceptual level*. A certain level of fuzziness exists around the concepts of entrepreneurs and entrepreneurship. Multiple related terms (policy entrepreneurs, political entrepreneurs, governance entrepreneurs, champions, advocates, or frontrunners) are in use and their interrelationships are not very clear, although Green (2017: 1473) suggests that two elements do seem to be present in most definitions: “First, policy entrepreneurs try to effect change; second, they use their agency to do so.” But who could entrepreneurs be, and how could we model their decision to become active? And if entrepreneurship involves groups of actors—across different jurisdictional levels and across various life worlds (politics, the bureaucracy, business, civil society)—how can we understand the way such groups come about? Another challenge at the conceptual level is to do with the strategies that entrepreneurs apply. As indicated, headway has been made by distinguishing clearly between entrepreneurs and their strategies, but the next challenges are about connecting the use of strategies to effects, and to understanding how strategy use and effectiveness might be dependent on context (see Brouwer and Huitema, 2018, this issue; Boasson and Huitema, 2017; Green, 2017). One tough choice to make at the conceptual level is to define which kinds of effects to study. For instance, are we simply interested in the adoption of a policy or measures, or do we also regard the implementation phase and the effect of the law on the behavior of a firm? In addition, context is likely to affect the selection of strategies and the way such strategies create effects. So far, little systematic thinking has gone into conceptualizing the relationship between entrepreneurial strategies and context. In connection with this, we asked the contributors to this special issue to reflect on the first guiding question for the special issue: *How to better conceptualize of governance entrepreneurship at the local and regional levels, and its broader effects on climate policies, be they local, regional, national or global?*

The second set of issues is *methodological*. The key issue here is attribution—to which degree can we reliably attribute adoption and implementation of climate governance to the presence and strategies of entrepreneurs, and how can we conclude that the involvement of entrepreneurs was a necessary or sufficient condition for policy change, of whichever kind and in whichever stage of the policy process? Careful and transparent process tracing is necessary and the limits to the agency perspective should be acknowledged (luck, coincidence, and unexpected events also do play a role). Also, one should be careful about the effects that research on entrepreneurship can have on reality itself—quite a few people generally take pride in being an entrepreneur (and even better *the* entrepreneur)—but studying them under the flag (and related terms such as champions, for instance) can mean that people start giving in to the inclination to overestimate their roles. The focus on successful cases is also potentially problematic, in multiple ways. One is very simple: if success was attained in one setting at one particular moment that does not mean that the approach tried there will always lead to success (Duineveld et al., 2009). Perhaps 100 other attempts to apply the same strategies were made, and none was successful. In other words, from a methodological perspective, it is probably also necessary to study unsuccessful attempts at entrepreneurship (see also Boasson and Huitema 2017). But even the labels success and failure should be applied with care because what is seen as a success may change over time (Van Assche et al. 2012). Applying either label presents problems from a methodological perspective. This is because successful or even heroic stories of policy change might be claimed by many, but failures might be claimed by no one. Another issue that complicates understanding is the balance between direction and coincidence. It is certainly true that entrepreneurs do not fully control the context that they operate in, but how do we separate pure luck from effective planned interventions? In connection with this, the second guiding question behind this special issue is: *Which methods are available for the researcher who want to analyze the existence and strategies of local and regional governance entrepreneurs, how can attribution problems be overcome, and which methods are suitable for the production of valid and reliable knowledge about entrepreneurship?*

In terms of *empirical knowledge gaps*, there are still plenty. One limitation in our understanding of entrepreneurship is geographical—there is less insight into how climate governance entrepreneurship operates in developing countries compared to developed countries, for instance—even though Huitema and Meijerink’s work (2009) seems to indicate that the dynamics might be quite different, with developing countries adopting more policy changes which are carried to them by exogenous entrepreneurs rather than endogenous ones, and which subsequently often become contested (compare Biesenbender and Tosun, 2014). Most of the literature focuses on policy adoption and so we do not know much about the role of entrepreneurs in later stages of the policy cycle, although Huitema and Meijerink (2009) propose that entrepreneurs are likely to turn into defenders of the status quo as soon as their ideas become implemented. Most of the gaps in our empirical knowledge follow from the limitations to the literature that we described before. This is logical because when the conceptualization of policy entrepreneurship does not reflect the importance of context for instance, we will not expand our knowledge of this factor. This means that the most pressing questions are in line with the conceptual gaps we just identified. Therefore, the third and fourth guiding questions for this special issue are as follows: *How can these entrepreneurs in local and regional climate governance be identified and characterized, in which situations are they active, what motivates them, and which strategies do they employ under which circumstances?* And *What are the effects of entrepreneurial activity at the local and regional levels on climate governance, in terms of the introduction of new problem definitions, new goals, and new strategies, the spread of such inventions across jurisdictions, and in terms of establishing and creating innovations with real impact, and how do these effects differ for variations in contexts?*

Keeping these key issues with the literature and these four guiding questions in mind, we now move to a brief summary of the various contributions that were made (Section 4) and we subsequently discuss the overall results of the special issue, harking back to the guiding questions (Section 5).

## Contributions to this special issue

As editors, we are pleased to have a wide range of contributions, all concerned with local and regional governance entrepreneurs. Some authors have taken a micro perspective and zoomed in on specific individuals and how they try to innovate climate governance, whereas others have focused on entire jurisdictional entities (municipalities, states) and how these help change current approaches. Most contributions focus on entrepreneurs in the public sector, but there are some who zoom in on private actors. The objects that these entrepreneurs try to influence also vary widely. Some of them are intent on influencing local and regional decisions, whereas others use their local background to aim at influencing national or global climate policies. Each of the contributions addresses at least one of the guiding questions behind this special issue. We summarize each of the contributions briefly.

*Stijn Brouwer* and *Dave Huitema* (this special issue) contribute to three of the four guiding questions: greater conceptual clarity about what it exactly is that entrepreneurs do in terms of their strategies (guiding question 1), and a rigorous methodology for investigating the incidence of such strategies, the reasons why governance entrepreneurs use them (guiding question 2), and the effects of context on effectiveness. They find indications that entrepreneurs have a personal inclination towards certain strategies, and this inclination seems correlated with several background factors such as education and also gender. They assess the incidence and efficacy of each of these strategies (and several sub categories for each type) by means of a *census study*—a type of research that seeks to locate the entire population for a certain target group rather than a sample. Their empirical analysis was focused on water management in the Netherlands. They attempt to identify all entrepreneurs working in this field. Through a *mixed method approach*—essentially a survey amongst all entrepreneurs and follow-up interviews and focus groups with specific individuals—they attempted to study which strategies are most suitable in a range of contextual conditions.

*Karen Anderton* and *Joana Setzer* (this special issue) also focus on regional government and they attempt to understand how the states of California and Sao Paulo have affected their respective national government, but also climate governance at the international level (guiding questions 3 and 4). In doing so, they do not so much highlight the role of highly active individuals—but they rather emphasize how the two jurisdictions have operated in their entirety (collective entrepreneurship). They suggest that not much is known currently about the innovative role of subnational government, and that this role is neither well understood. They succeed particularly well in showing how both California and Sao Paulo are considered leaders internationally—bypassing hesitating national governments. They do have quite an impact and their innovations are impressive; Sao Paulo was the first jurisdiction in a developing country to set binding emission reduction targets, and California is widely seen as a pioneer in the realm of emissions trading and developing comprehensive climate law. Both states project their ideas at the national state—showing how climate governance could look like, and solving legal puzzles (and obstacles) along the way. They network with each other and with other regions, and especially, California has gone so far as to create transnational governance initiatives by linking its emissions trading scheme with that of other regions (such as Quebec).

*Tobias Renner* and *Sander Meijerink* (this special issue) also focus on transnational policy entrepreneurship, specifically in the Rhine river basin across the German-Dutch border. Cross border collaboration is promoted by EU-water law, for instance in the context of developing river basin management plans, and the question that Renner and Meijerink 2018, seek to answer is which strategies policy entrepreneurs on both sides of the border employ to incorporate climate adaptation issues in such plans (guiding question 4). Their analysis suggested that out of some 60 public officials involved, 5 could be seen as policy entrepreneurs. They find that entrepreneurs use a range of strategies, but they do note that for the majority of them, strategy selection is a process of “trial and error,” which suggests that entrepreneurs are fallible humans too. A remarkable obstacle to transboundary collaboration is located in the differences in which climate change is being framed in both countries—whereas Germany approaches climate change issues as a broad set of interrelated issues, their Dutch counterparts focus largely on water issues. The policy entrepreneurs involved initiate and direct such projects—and one of their most delicate tasks is to manage the set of actors involved (some will be activated, others not)—they do so with a keen awareness of the institutional and organizational mandates of parties involved. However, Renner and Meijerink suggest that for the moment, any policy change reflecting climate change problems is still very much a paper tiger, without much action on the ground. They offer several contextual explanations. The first is that when it comes to water issues and climate change, the Netherlands is ahead of Germany. In addition and interestingly, they suggest that especially measures that deviate from current practices meet with greater resistance, meaning that such innovations take longer. Finally, they suggest that climate adaptation competes for attention with climate mitigation—which means that in the case of Germany, much attention has been devoted to mitigation (specially the energy transition) rather than adaptation.

*Sarah Giest* (this special issue) focuses on the case of Swedish wind coordinators—four people appointed for one day per week throughout the country to stimulate the constructions of wind turbines, on shore and off shore. The case is somewhat different from others in this special in the sense that these entrepreneurs have been appointed at the national level to assist at the local level. They are carefully selected, for instance from the perspective of previously existing connections with the regions in which they work, and relevant experience in the public sector, but they are not people who have come to the forefront at their own initiative (guiding question 3). In this sense, this is an interesting case of willful or directed use of entrepreneurship at the stage of policy implementation. Giest asks whether the wind coordinators are able to use this position to undertake entrepreneurial activities that potentially go beyond the indicated structural position assigned by government. She uses the typology of entrepreneur activities that was developed by Roberts and King (1991), distinguishing between four types of activities: creative, strategic, mobilization and execution, and lastly administrative and evaluative. She subsequently analyzes how the four wind coordinator positions operated (guiding question 4). The coordinators initially focused quite strongly on dissemination and mobilization—communicating advantages and disadvantages of wind turbines to local communities, and setting up demonstration projects. In addition, and somewhat later, they started engaging in a more strategic action as they started pleading for institutional changes—notably a simplification of planning permission procedures, and abolishment of taxes on the income that wind cooperatives make from wind turbines. The wind power coordinators are largely regarded as successful, but they have not achieved all they have aimed for. It took some time after they were appointed before they started to aim push above their weight. Over time, the coordinators re-defined their activities and moved beyond mobilization and execution activities to creative/intellectual and administrative/evaluative activities. Their entrepreneurial efforts were particularly aimed at changing administrative processes connected to setting up of wind energy and the tax systems. While the former has been altered somewhat in line with the ideas of the coordinators, this has not happened with taxation.

*Rachael Shwom* and *Analena Bruce* (this special issue) focus their analysis on the way American NGOs are influencing energy efficiency standards for appliances; their analysis covers the year 1972–2006 but goes beyond at several moments. From a methodological perspective (guiding question 2), this offers very interesting prospects to trace the involvement of policy entrepreneurs over time, under very diverging political and institutional conditions. They analyzed a wide range of events (from court cases to conferences) in that period and found that more than 1200 organizations had participated. A network analysis suggested 15 NGOs were relatively central to the events in this emerging field—and the authors were able to extensively discuss their strategies with 11 of them. Aided by the long time horizon of their analysis, Shwom and Bruce are able to show that through persistence and creativity in framing, the NGOs were able to develop an entire new “system of meaning” that revolves around the notion of “market transformation.” Helping our conceptual understanding (guiding question 1) and increasing our empirical insights into the application of strategies (guiding question 3), Shwom and Bruce 2018, pay extensive attention to the context in which entrepreneurs operate. The process of introducing new norms or regulations is so cumbersome in the USA (it can take up to 15 years to complete) that NGOs are effectively forced to work with their “opponents”—the industry. They show that industry is willing to collaborate only under the right political circumstances—when there is a more progressive government in power in Washington, which is willing to entertain more ambitious targets and regulation. In addition, the NGOs managed to organize forums for exchange of thought—particularly a set of conferences around energy efficiency, which—over the course of time—helped create a new organizational field. They also suggest that the creation of alternative institutions such as the ones around “pre negotiated standards” (norms that NGOs and business can agree on ahead of formal government standards) helped pilot additional institutional innovation by showing public and private regulators (government and voluntary certification bodies) what the norms could look like. A final and interesting observation emanating from this paper is that working with businesses in the particular context of the US efficiency standards regulation process requires entrepreneurs to behave like “honest brokers”—meaning that they must be seen as credible, taking the interests of business to heart, and knowledgeable about where the technology stands. They also observe that businesses are interested in working with NGOs because they can confer (or withhold) green legitimacy to products.

*Alexandra Mallett* and *David Cherniak* (this special issue) explore various contextual factors that influence the selection and effectiveness of entrepreneurial strategies, therewith contribution to guiding question 1 about the conceptualization of policy entrepreneurs. They draw on a study towards the development of new energy policies in subnational jurisdiction in the Canadian Arctic. Their study shows that institutional factors deserve attention in the analysis of policy entrepreneurs and their strategies. The institutional factors shape the conditions in which entrepreneurial activities are undertaken. Institutions can stimulate particular strategies, while delimiting the effects of other strategies. Institutional factors are important for understanding the local governance context, but also for analyzing how local decision-making arenas are embedded in wider governance context, e.g., by shaping the room for local autonomy or defining the possibilities for financial incentives from higher-level governments. In addition, Mallett and Cherniak 2018, argue that a policy entrepreneur is often a collective of actors, both public and private, that together develop and implement new policies, an insight that warrants more attention to the network of actors involved in climate policy innovations. A main argument of the paper is that policies that fit local circumstances and cultures are more likely to be adapted. Such policies can be found more easily if local actors are included in decision-making arenas and if policy entrepreneurs actively look for possibilities to link climate innovation with local issues and local ways of thinking. The paper for example points to the relevance of a sense of ownership that helps to ensure a more meaningful involvement of local actors in the process of finding new climate policies. The study indicates that particular institutions in the Canadian Artic, such as modern treaty and devolution agreements, do indeed facilitate local involvement and ownership and are therefore important in understanding the role of policy entrepreneurs and the effects of their strategies.

## Revisiting the guiding questions, ideas for future research

**Fig. 1:**
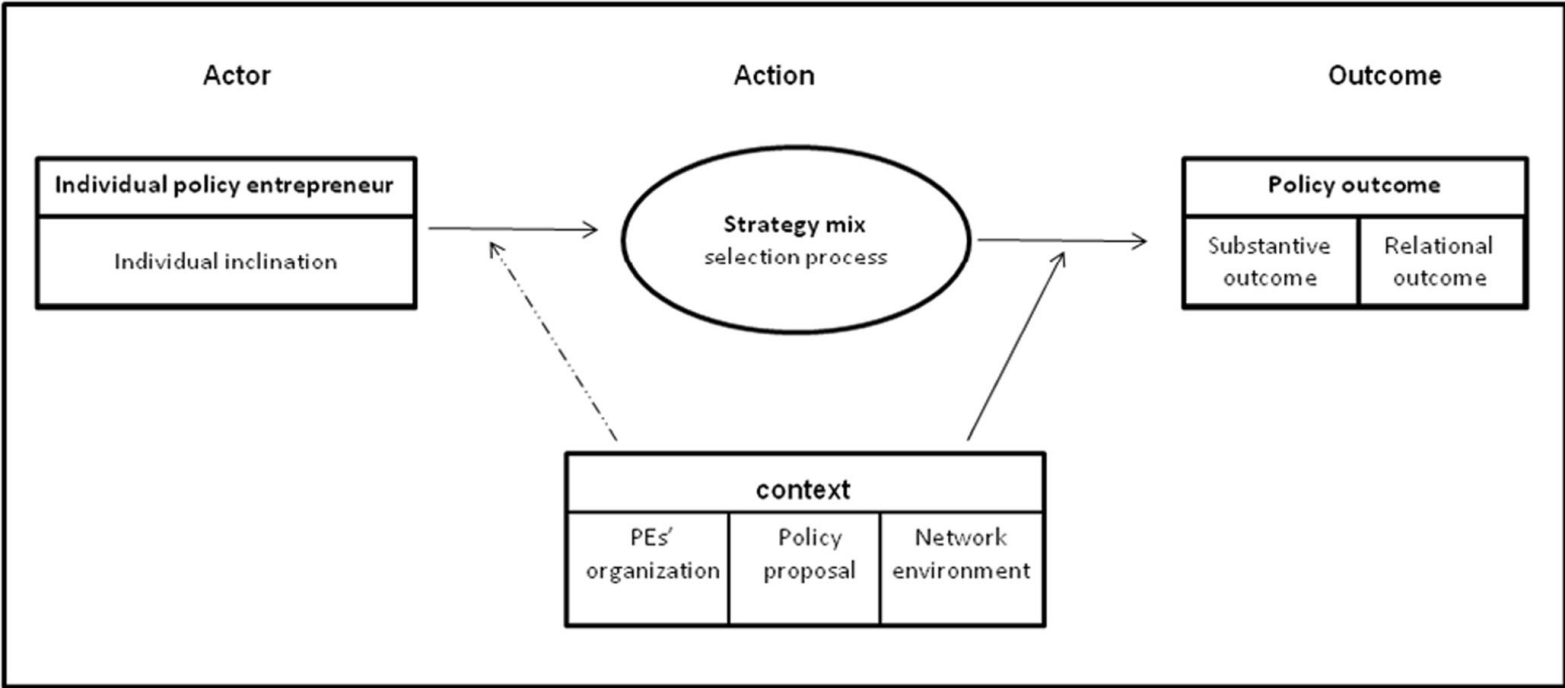
Conceptual scheme denoting entrepreneur activity. From: Brouwer and Huitema (this special issue)

Four questions drove our special issue. We discuss the most important findings emanating from the impressive amount of academic work showcased in the special issue by revisiting each question and highlighting the most valuable insights acquired through the various contributions. The first guiding question was “How to better conceptualize of governance entrepreneurship at the local and regional levels, and its broader effects on climate policies, be they local, regional, national or global?” The conceptual model that is presented by Brouwer and Huitema (this special issue) is worth reproducing as it will also help us in structuring the findings from the various other contributions to the special issue (Fig. 1).

At the heart of the conceptual frame is a set of strategies that an entrepreneur might select to affect change. The selection of strategies is influenced by a mix of personal inclinations and a selection of contextual factors—which include the support from the home organization of the entrepreneur, the nature of the policy proposal, and the network environment of the entrepreneur. The application of strategies produces outcomes, which can be of a substantive or a relational nature—the first type of outcome refers to the level of success achieved in terms of policy change and the second outcome is to do with the consequences that strategy application has for the position of the entrepreneur in the network.

In terms of the types of strategies, Brouwer and Huitema distinguish between attention- and support-seeking strategies, linking strategies, relational management, and arena strategies. The various other typologies used in the contributions to this special issue align quite well with this. Shwom and Bruce (this special issue) distinguish between framing, trust building, and field structuring events and these fit under attention- and support-seeking, relational management, and arena strategies respectively. Giest 2018, (this issue) talks about dissemination and mobilization and setting up demonstration projects, which would all fall under the heading of attention- and support-seeking strategies. She also notices how wind power coordinators in Sweden later engaged in venue shopping, as they detected that not all issues could be addressed effectively at the local level.

Through the conceptual scheme, we also get a clearer understanding of the relevant considerations going into strategy selection—the mix of personal inclinations on behalf of entrepreneurs and *contextual settings* such as the nature of the proposal (how radical is it), the network context, and internal support for the entrepreneur in the organization that they work for. There are various indications that this typology of contextual factors captures a lot, but not all, of factors that determine the use of strategies. Renner and Meijerink (this special issue) confirm that the *nature of the proposal* is relevant, and particularly the degree to which it constitutes a radical departure from the status quo—they suggest that more radical proposals will create more opposition, and hence pushing them through will have greater relational consequences. They do not suggest how this affects the choice of strategies exactly, because their contention is that strategy selection is in practice a matter of trial and error. Shwom and Bruce (this special issue) highlight the importance of networks, and specifically the perceptions that actors have of each other within these networks. In doing so, they actually connect two contextual variables, the network with the nature of the proposals that entrepreneurs can make—“overly radical” proposals will affect the standing of environmental NGOs with businesses, whereas economically and technically, sound proposals could result in a more welcoming stance.

The conceptual scheme does miss various elements of the context which the other authors point at—notably the institutional, economical, and the political contexts, which are receiving quite a lot of attention in the contributions by Shwom and Bruce (this special issue), Mallet and Cherniak (this special issue), and Renner and Meijerink (this special issue). In institutional terms—Shwom and Bruce suggest that it matters whether a policy field is very judicialized—they argue that when most decisions on policy must be taken by the courts, parties (natural opponents such as companies and environmental NGOs) have an incentive to collaborate. But whether they really do also depends on the degree to which NGO endorsement is of interest to market parties in an economic sense. It also depends on the questions whether political leadership is interested in energy saving—if it is, companies have greater incentives to produce innovative standards together with environmental NGOs than in a situation where politicians have no interest or when they oppose more stringent standards. Mallet and Cherniak (this special issue) point to the particular institutional context in Artic Canada that stimulates stakeholder involvement and local ownership, both important for developing novel climate policies. Renner and Meijerink (this special issue) argue that priorities of politicians (in this case adaptation or mitigation) affect the space for entrepreneurial activity. These studies show that context can mean many things and that which factors are most relevant depends on that context.

Finally, in terms of the possible outcomes from the application of entrepreneurial strategies, the model captures most but not all discussions in this special issue. Most studies pay attention to the substantive policy outcomes that the entrepreneurship can bring, but the fact that relational outcomes matter is also confirmed in some of the contributions to the special issue. Notably, Shwom and Bruce (this special issue) suggest that environmental NGOs need to win the trust of the electrical appliance industry, and that they can do this by presenting proposals that make sense from a technical and economical perspective. Brouwer and Huitema (this special issue) suggest that policy entrepreneurs—at least in the Dutch context—do not play a single game, but rather a *series of games*. If the strategy is to exclude certain parties, this could affect relationships later on—this shows that dependencies between actors mean that relational outcomes weigh heavily in strategy selection. The model seems to miss various other outcomes from the work of entrepreneurs—which we could perhaps label institutional outcomes. Shwom and Bruce (this special issue) for instance show how environmental NGOs deliberately create new fields by organizing events where a running conversation can take place on policies and standards that could apply in the future. In a similar vein Mallet and Cherniak show how energy charrettes, venues for bringing a wide range of actors together, act in a similar way. When done well, such meetings could create a constituency of motivated actors—who can set common goals or work to increase acceptance for some of these goals.

The second guiding question for this special feature was “Which methods are available for the researcher who want to analyze the existence and strategies of local and regional governance entrepreneurs, how can attribution problems be overcome, and which methods are suitable for the production of valid and reliable knowledge about entrepreneurship?” We mention a few examples. First of all, the selection of a long time span does appear to add to the analysis of what entrepreneurs do and what they can achieve. Shwom and Bruce (this special issue), with their focus on efficiency norms for appliances, trace the discussions for the past 34+ years. Their analysis reveals how entrepreneurship must in some cases (but we do not know how many) be exercised over a very long time in order to achieve progress. It also shows that progress is never safe or secure, and that the political mood changes considerably over time—sometimes creating opportunities, sometimes bringing obstacles. We speculate that the notion of institutional outcomes (the fact that NGOs can establish a field) would have gone unnoticed if the time frame had been shorter. The notion of a *census* as described by Brouwer and Huitema (this special issue) is also an interesting option to be considered for more research in the future, even if it is a demanding approach (phoning all heads of the local and regional bureaucracy, asking them to identify employees who meet the profile, and then confirming with these employees whether they agree means 1000 s of phone calls needed to be made). The fact that all entrepreneurs in a certain field (in this case Dutch water management) are included in a study means that the validity of the outcomes is pretty much secured—although questions can still be asked about the transferability of findings to other fields that remains to be seen. The logical suggestion is then to start doing comparative analysis of entrepreneurs who work in different fields. One matter that could potentially warrant comparative analysis is the exceptionally high number of entrepreneurs that Brouwer and Huitema identified, which really does not align with much of the existing work that focuses on a handful of individuals in particular case studies. The very same paper also shows some of the limitations of the census method—at least the way it was applied here. Because the survey focused on their experience in general, it is hard, if not impossible, to check outcomes of the application of strategies in specific case studies and the basis for certain conclusions on effectiveness is relatively dependent on self-reporting. A combination with actual case study analysis would have probably helped to further cement the conclusions.

The third guiding question was: “How can these entrepreneurs in local and regional climate governance be identified and characterized, in which situations are they active, what motivates them, and which strategies do they employ under which circumstances?” The contributions to the special issue do contain multiple indications that are relevant for these issues. In essence, all entrepreneurs described in this special issue go beyond their ordinary tasks in seeking policy change. Remarkably often—at least in the contributions that explicitly discuss it here—the motivation for becoming active is to do with the mission of an organization. Entrepreneurs become active because the mission of their organization requires action in the climate domain. In one instant, the entrepreneurs have actually been tasked to bring policy change (see Giest 2018). The degree to which motives such as competition between authorities play a role in this remained relatively unexplored here. The clearest findings about strategy selection in this special issue came from Brouwer and Huitema (this special issue), who concluded that—at least in the Dutch water management setting—(1) policy entrepreneurs tend to avoid alarming messages about potential impactful events; (2) they assemble coalitions that contain all necessary parties but that they exclude some parties, especially vehement opponents so as to avoid complexity; and (3) they care deeply about content but even more about maintaining relationships—which sometimes leads them to accept compromise or even temporary defeat, in the hope of making up later on.

The fourth and last guiding question was “What are the effects of entrepreneurial activity at the local and regional levels on climate governance, in terms of the introduction of new problem definitions, new goals, and new strategies, the spread of such inventions across jurisdictions, and in terms of establishing and creating innovations with real impact, and how do these effects differ for variations in contexts?” Renner and Meijerink (this special issue) suggest that entrepreneurs have a hard time overcoming the differences between the Netherlands and Germany, both in the sense of the priority that climate change adaptation gets (high in the Netherlands, low in Germany) and in the sense of the governing cultures (which are said to be more hierarchical in Germany, thereby offering less space for entrepreneurs). Shwom and Bruce (this special issue) suggest that over time, especially through smart framing and by the organization of conferences and meetings, certain NGOs have managed to develop a constituency around the notion of more efficient household appliances. They furthermore detect possibilities for such NGOs, ahead of formal government regulation, to develop performance standards with the industry. In addition, they can help companies market greener appliances by lending their approval to claims about better performance. Anderton and Setzer (this special issue) highlight the various ways in which regional actors like Sao Paolo and California develop new policies and push them at the global level.

Overviewing the various conclusions we can draw on the basis of this special issue, we wish to suggest that academic work on entrepreneurship in climate governance is worthwhile and is indeed generating insight into the reasons why and how new policies are developed. We identify the following opportunities for future academic work in this field, organized according to the distinction between conceptual, methodological, and empirical work.

Regarding the *conceptual work* that still needs doing, it is our conviction that much more interaction is possible between scholars who work on entrepreneurship and the scholars who work on specific aspects of policy innovation. When it comes to the invention of new governance approaches for instance, collaboration with those approaching the invention of “new governance technologies” from the perspective of Science and Technology Studies could be attractive. Voss and Simons (2014) for instance detail how scientific communities interact with practitioners to produce new policy instruments such as emissions trading—important events in this invention process include trials that deliver proof of principle, the standardization of such technologies, and the spotting of business opportunities in connection with technology, which create a constituency for the instrument. One line of research could focus on the involvement of governance entrepreneurs in each of these steps. When it comes to diffusion studies, there is a wealth of opportunities for connecting the work on entrepreneurship to it. Diffusion scholars (see, e.g., Biesenbender and Tosun 2014) work with relatively crude model that hinges on the distinction between internal and external reasons for adopting new policies. There are plenty of signs (see Huitema and Meijerink 2009) that such a distinction is untenable in a world where policy entrepreneurs navigate between governance levels and play a key role in both up- and downloading ideas between such levels, and the subsequent implementation in relevant jurisdictions. In the diffusion literature on climate governance for instance, it is shown that in countries that accept international commitments, opponents seek to slow down implementation (Biesenbender and Tosun, 2014) and “counter-entrepreneurs” might be involved in this process.

Regarding the *methodological work* that lies ahead of us, we believe that it is important to perform more mixed method work, for instance to combine large-scale surveys with case studies. Further longitudinal studies can provide useful insights into the contingent interplay between entrepreneurial strategies, effects, and contexts. We believe that entrepreneurship studies would benefit from more systematic comparisons between different issue areas, for instance comparing climate governance with health care, education, or other governance areas. Further, we call for entrepreneurship studies that compare localities and regions that have adopted and implemented ambitions climate policy and those that have not.

Finally, in terms of empirical evidence, this special issue has produced some insight, but there is still a world to win. One of the more pressing issues is probably the interaction between public and private entrepreneurship—this special issue has been relatively light on analyses focusing on private actors, but Green (2013) argues that private regulation will become increasingly important in the future and this will happen in a process that involves both public and private actors. Where and how entrepreneurs play a role in this, and how they might move between the sectors, or build coalitions across them, is still relatively unknown. Fortunately for us, entrepreneurs are already at work creating new policies, diffusing them, and making sure they have an effect, so the universe that can be studied is expanding.
